# Nonlinear processes reinforce extreme Indian Ocean Dipole events

**DOI:** 10.1038/srep11697

**Published:** 2015-06-26

**Authors:** Benjamin Ng, Wenju Cai, Kevin Walsh, Agus Santoso

**Affiliations:** 1 CSIRO Marine and Atmospheric Research, Aspendale, Victoria, Australia; 2 School of Earth Sciences, University of Melbourne, Parkville, Victoria, Australia; 3 Australian Research Council Centre of Excellence for Climate System Science, University of New South Wales, Sydney, New South Wales, Australia

## Abstract

Under global warming, climate models show an almost three-fold increase in extreme positive Indian Ocean Dipole (pIOD) events by 2100. These extreme pIODs are characterised by a westward extension of cold sea surface temperature anomalies (SSTAs) which push the downstream atmospheric convergence further west. This induces severe drought and flooding in the surrounding countries, but the processes involved in this projected increase have not been fully examined. Here we conduct a detailed heat budget analysis of 19 models from phase 5 of the Coupled Model Intercomparison Project and show that nonlinear zonal and vertical heat advection are important for reinforcing extreme pIODs. Under greenhouse warming, these nonlinear processes do not change significantly in amplitude, but the frequency of occurrences surpassing a threshold increases. This is due to the projected weakening of the Walker circulation, which leads to the western tropical Indian Ocean warming faster than the east. As such, the magnitude of SSTAs required to shift convection westward is relatively smaller, allowing these convection shifts to occur more frequently in the future. The associated changes in wind and ocean current anomalies support the zonal and vertical advection terms in a positive feedback process and consequently, moderate pIODs become more extreme-like.

Indian Ocean Dipole (IOD) events occur on interannual time scales with positive Indian Ocean Dipole events (pIODs) associated with cold sea surface temperature anomalies (SSTAs) over the eastern Indian Ocean (IO) and warm SSTAs to the west^1^,^2^. The pattern is opposite for the negative phase of the IOD (nIOD). During pIODs, East Africa and India experience wetter than normal conditions whilst drier conditions occur over Indonesia and Australia^3^,^4^,^5^,^6^,^7^. Since 1960, there have been three extreme pIOD events: in 1961, 1994, and 1997^8^. These extreme episodes differ from moderate pIODs in that the cold SSTAs in the east are larger in amplitude, and extend further westward than normal. This generates anomalously dry conditions along the equatorial IO, pushing the centre of atmospheric convergence further west. When this occurs, a positive feedback process begins whereby the mean westerly winds are weakened and equatorial upwelling (downwelling) Kelvin (Rossby) waves are generated^8^. This leads to stronger upwelling off the Sumatra-Java coast whilst the thermocline deepens in the western tropical IO which further strengthens the zonal temperature gradient and the anomalous easterly winds. This positive feedback along the equator allows pIOD events to become extreme and thus makes their impacts considerably stronger^8^, with damaging floods and malaria outbreaks occurring in Eastern Africa and the Indian subcontinent^4^,^9^,^10^,^11^ whilst Australia and Indonesia experience severe drought^2^,^12^,^13^.

There are several atmosphere-ocean feedbacks that are associated with the IOD^14^. One of these is a positive wind-evaporation-SST feedback where cold SSTAs over the eastern IO lead to stronger winds, which in turn increases evaporation and thus reinforces the cold SSTA. Another reinforcing factor is the Bjerknes feedback, involving interactions between the thermocline depth, SSTAs, and the wind. This feedback is the primary cause of SST skewness in the eastern IO in models^15^,^16^. The SST-cloud-radiation process is a negative feedback which damps pIODs more strongly than nIODs^6^,^15^,^17^. Finally, there is a nonlinear dynamic heating (NDH) process which is composed of three processes: anomalous zonal, meridional, and vertical advection of the temperature anomaly^14^,^17^,^18^,^19^. This process has been shown to reinforce pIODs by cooling the eastern IO but damps warm SSTAs that are associated with nIOD events^8^,^17^,^18^,^19^. Over the tropical IO, the NDH term is primarily composed of nonlinear zonal and vertical advection while the second term, nonlinear meridional advection, does not play a major role in the development of either moderate or extreme pIODs^17^. The NDH process plays an important role in the generation of extreme pIODs^8^, providing additional cooling of the cold SSTA in the east and advecting this cold anomaly from the east, towards the tropical western IO.

Nonlinear dynamic heating and coupled wave propagation via Kelvin and Rossby waves are both important processes for the development and intensification of extreme events^8^. This is because these two processes are linked in a positive feedback where a stronger negative zonal (pIOD) temperature gradient reinforces nonlinear advection and also the anomalous zonal winds. Stronger nonlinear advection helps to extend the cold SSTAs along the equator, pushing the centre of convergence westward. Simultaneously, the anomalous zonal winds can generate upwelling (downwelling) Kelvin (Rossby) waves which cool (warm) the eastern (western) equatorial Indian Ocean, reinforcing the anomalous zonal temperature gradient^2^,^8^,^20^,^21^.

Under global warming, the tropical IO undergoes a mean state change that is driven by a weaker Walker circulation^14^,^22^,^23^,^24^,^25^. This weakens the mean westerly winds and generates a pIOD-like warming pattern where the western IO warms faster than the east, but when referenced to a changing mean state, there is no noticeable change in the amplitude or frequency of pIOD events^14^,^24^. However when referenced to the fixed present-day mean, a recent study^8^ showed that the frequency of extreme pIOD events is expected to increase by almost three times under increasing greenhouse gases. As extreme pIODs have the potential to impact millions of lives, it is important to understand the processes involved in their generation and the causes of the expected increase under global warming. NDH has been suggested as a critical process in generating more frequent occurrences of extreme pIODs under greenhouse warming^8^ but an explicit heat budget analysis of the simulated events has not been done. Heat budget analysis allows the relative roles of ocean advection and surface heat flux to be assessed. This can reveal the physical processes associated with extreme pIODs and the mechanisms behind the projected increase in frequency. In this present study, the surface heat budget of simulated extreme pIOD and nIOD events is analysed with the aim of further clarifying the role of the NDH process.

## Results

**Figure 1:**
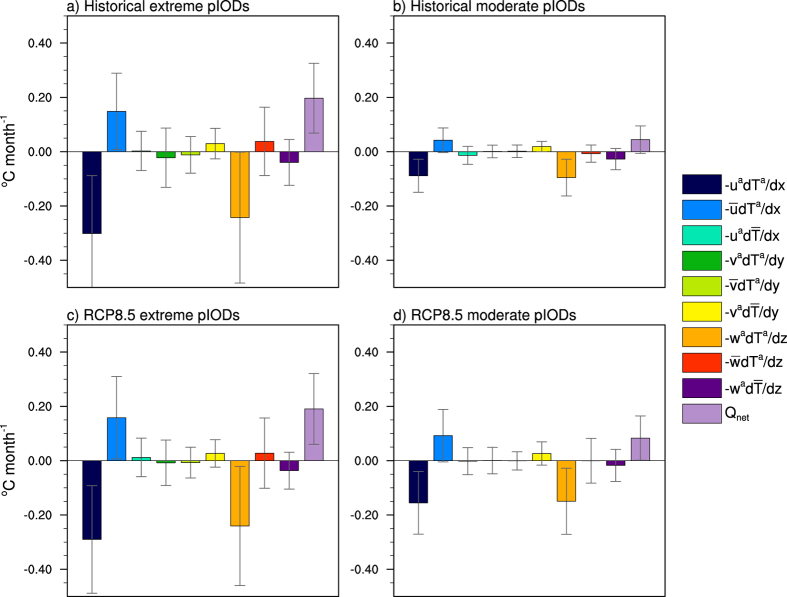
Heat budget analysis of extreme and moderate pIOD events in CMIP5 models. (**a**) Multi-model ensemble averaged historical (1911–2005) SON heat budget components for extreme pIODs. (**b**) As in (**a**) but for moderate pIODs. Moderate pIODs are when the Dipole Mode Index is greater than 0.75 standard deviations but not an extreme event. (**c**), (**d**) As in (**a**), (**b**) respectively but for the RCP8.5 period (2006–2100). The error bars indicate 1 standard deviation of the multi-model ensemble. The heat budget components are averaged over the equatorial IO (60°E-100°E, 5°S-5°N). Table S2 provides a description of each heat budget term. All plots were generated in NCL.

Heat budget analysis of 19 models (see Table S1) from phase 5 of the Coupled Model Intercomparison Project (CMIP5) reveals that nonlinear zonal and vertical advection dominate during historical extreme pIODs (Fig. 1a; for definitions of the individual terms, see the Heat budget analysis section in the Methodology and Table S2). For non-extreme pIODs, the magnitude of these two nonlinear advection terms is substantially weaker, almost one-third that of extreme events. The other heat budget terms are also weaker due to the reduced forcing but the dominance of the nonlinear zonal and vertical advection shows that they still play an important role in generating and sustaining moderate pIODs (Fig. 1b). The strong influence of nonlinear zonal and vertical advection during extreme pIOD events is also seen in data from the European Centre for Medium-Range Weather Forecasts – Ocean Reanalysis System 3 (ECMWF ORA-S3; Fig. S1), suggesting that the CMIP5 models are able to capture the dynamics associated with extreme events. Under increasing greenhouse gases, nonlinear zonal and vertical advection remain important contributors to the cooling of the equatorial IO during extreme pIODs (Fig. 1c) but their amplitude does not change noticeably from the historical simulation. In contrast, moderate pIODs exhibit a distinct increase in amplitude for the two terms (Fig. 1d).

**Figure 2:**
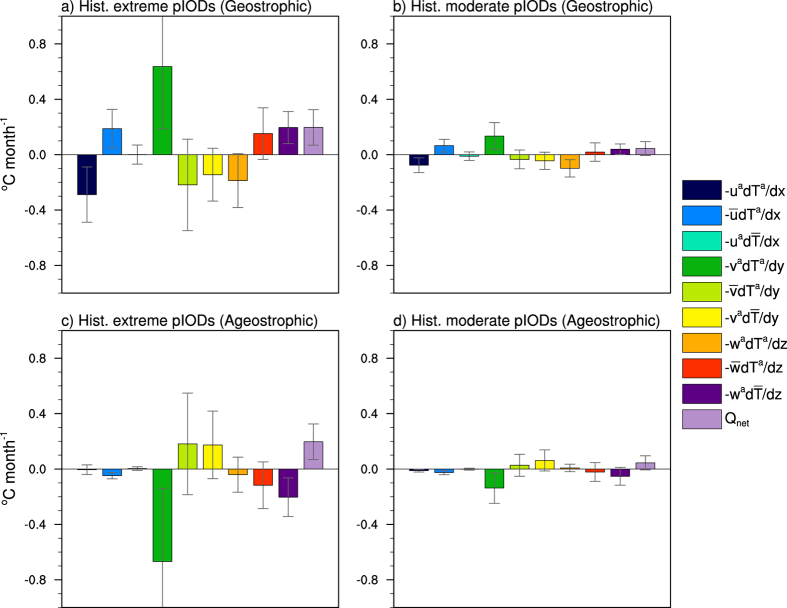
Historical geostrophic and ageostrophic heat budget analysis of extreme and moderate pIOD events in CMIP5 models. (**a**) Multi-model ensemble averaged historical (1911–2005) SON heat budget components for extreme pIODs using only geostrophic currents. (**b**) As in (a) but for moderate pIODs. (**c**), (**d**) As in (**a**), (**b**) respectively but for ageostrophic currents only. The error bars indicate 1 standard deviation of the multi-model ensemble. The heat budget components are averaged over the equatorial IO (60°E-100°E, 5°S-5°N). All plots were generated in NCL.

The advective terms of the heat budget analysis can be further decomposed into the geostrophic and Ekman components using zonal and meridional surface wind stress to derive the Ekman velocities. The geostrophic velocities can then be approximated by subtracting the ageostrophic component from the total ocean velocities. The results show that the geostrophic velocities dominate the nonlinear zonal and vertical terms (Fig. 2) of the total heat budget analysis but as the Coriolis parameter (  *f*  ) equals zero along the equator, there may be some uncertainties involved. For this analysis, the primary focus is on the total terms (i.e., the geostrophic plus ageostrophic terms as shown in Fig. 1) along the equator.

### Spatial structures associated with IOD events

As nonlinear zonal and vertical advections are strong during extreme pIODs, the cold SSTA in the east extends further west (Figs. S2a,c), assisting the generation of the equatorial positive feedback. This does not occur for moderate pIODs (Figs. S2b,d), moderate nIODs or strong nIOD events (Fig. S3). As there is no westward extension of the SSTA in the eastern tropical IO during these other events, the positive feedback involving equatorial Kelvin waves cannot be generated. Moreover these nonlinear terms act to damp nIODs, cooling the anomalously warm eastern IO instead of further warming it (Figs. S4–S6). Thus, NDH is a process unique to the generation of extreme pIOD events.

**Figure 3:**
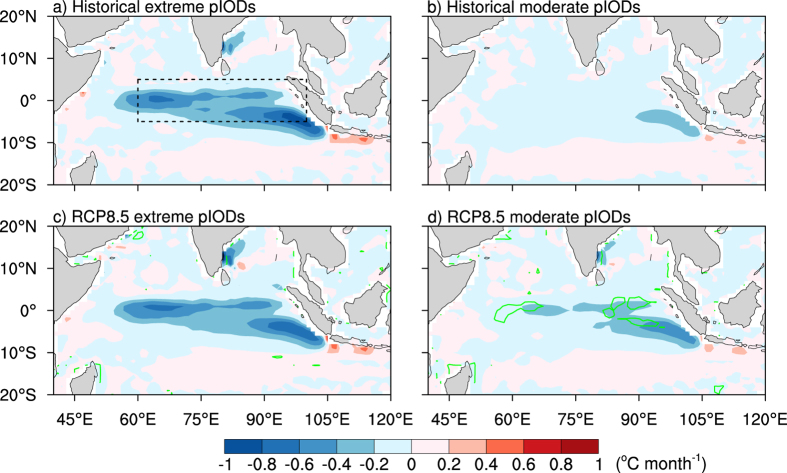
Nonlinear zonal advection during extreme and moderate pIOD events. (**a**) Map showing the multi-model ensemble mean historical SON nonlinear zonal advection term during extreme pIODs. (**b**) As in (**a**) but for moderate pIODs. (**c**), (**d**) As in (**a**), (**b**) respectively but for the RCP8.5 period. The dashed black box in (**a**) marks the equatorial IO region (60°E-100°E, 5°S-5°N) and the green contours in (**c**) and (**d**) denote where the difference between the historical and RCP8.5 periods is significant at the 95% confidence level. The significance is calculated using a two-tailed Student’s *t*-test. All maps were generated in NCL.

Figure 3a shows the multi-model ensemble grid-point nonlinear zonal advection for historical extreme pIOD events. Compared with Fig. 3c, it is clear that under global warming, there is no significant change in the nonlinear zonal advection term over the equatorial IO (60°E-100°E, 5°S-5°N). However, the importance of this term for the development of extreme pIODs can be seen by comparing it with that of moderate pIODs (Figs. 3b,d). For extreme pIOD events, nonlinear zonal advection induces cooling in the western and central equatorial IO (between 50°E-90°E). This allows the cold SSTA in the east to extend further westward (Figs. S2a,c), generating anomalously dry conditions along the equator and pushing the centre of convection further west, in contrast to the conditions during moderate events (Figs. 3b, S2b). As this occurs, a positive feedback process forms where the westward shift in convection drives a stronger anomalous negative zonal temperature gradient and anomalous easterly zonal winds^8^. These easterly zonal wind anomalies reduce the mean wind along the equator, generating upwelling Kelvin waves that strengthen the cooling in the eastern IO while the thermocline deepens in the west^8^. Consequently, the nonlinear zonal advection process is reinforced and this is conducive for extreme pIOD growth.

In contrast, during moderate pIOD events in the historical period (Fig. 3b), moderate nonlinear zonal advective cooling is limited to the eastern equatorial IO (between 90°E-105°E, 10°S-Eq.), and is thus not conducive for shifting convection westward or the ensuing positive feedback along the equator. However under increasing greenhouse gases, nonlinear zonal advection associated with moderate pIODs displays a considerable increase in cooling along the equator, parts of which are significant at the 95% confidence level (Fig. 3d). This would help to generate cold SSTAs and anomalously dry conditions that are typical of extreme pIODs and therefore, the nonlinear zonal advection process that occurs during moderate pIODs is becoming more extreme-like in a warmer climate. The importance of nonlinear zonal advection is further highlighted by the pattern associated with moderate and strong nIODs (Fig. S5). It is clear that for strong nIODs, there is no substantial westward extension of the nonlinear zonal advection term due to the cooling effect of the NDH process (Figs. S5a,c).

**Figure 4:**
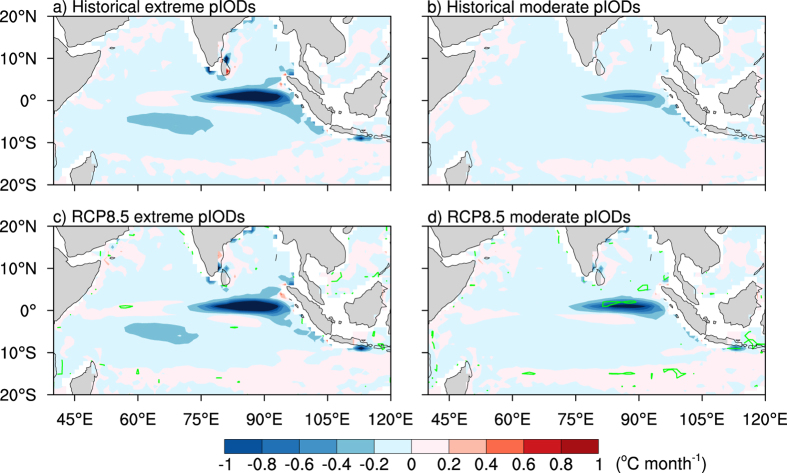
Nonlinear vertical advection during extreme and moderate pIOD events. (**a**) Map showing the multi-model ensemble mean historical SON nonlinear vertical advection term during extreme pIODs. (**b**) As in (**a**) but for moderate pIODs. (**c**), (**d**) As in (**a**), (**b**) respectively but for the RCP8.5 period. The green contours in (**c**) and (**d**) denote where the difference between the historical and RCP8.5 periods is significant at the 95% confidence level. The significance is calculated using a two-tailed Student’s *t*-test. All maps were generated in NCL.

Nonlinear vertical advection during extreme events also shows little change under greenhouse warming (Figs. 1a,c). The influence of this process is largely confined to the eastern equatorial IO (between 75°E-95°E), where anomalous upwelling advects anomalously cool subsurface water towards the surface (Fig. 4a). Over this region, the change between the historical and RCP8.5 periods is indistinguishable for extreme events (Figs. 4a,c). For moderate pIODs, there is an increase in amplitude over the eastern IO (Figs. 4b,d), and this suggests that nonlinear vertical advection associated with moderate pIODs is becoming stronger in RCP8.5, as is the case for the nonlinear zonal advection. Nonlinear vertical advection is an important process for extreme events as the cold temperature anomalies in the eastern IO that are reinforced by nonlinear vertical advection can then be advected westward. Furthermore, the extra cooling strengthens the zonal temperature gradient, leading to further weakening of the mean westerly winds. This assists the generation of the positive equatorial feedback involving upwelling (downwelling) Kelvin (Rossby) waves and therefore reinforces the development of extreme pIOD events. In contrast, nIODs are damped by nonlinear vertical advection along the equatorial eastern IO as indicated by the negative anomalies shown in Fig. S6. Not only does this damp the warm SSTA in the east, it also weakens the anomalous westerly winds and limits generation of the equatorial positive feedback.

### Processes governing nonlinear zonal and vertical advection

**Figure 5:**
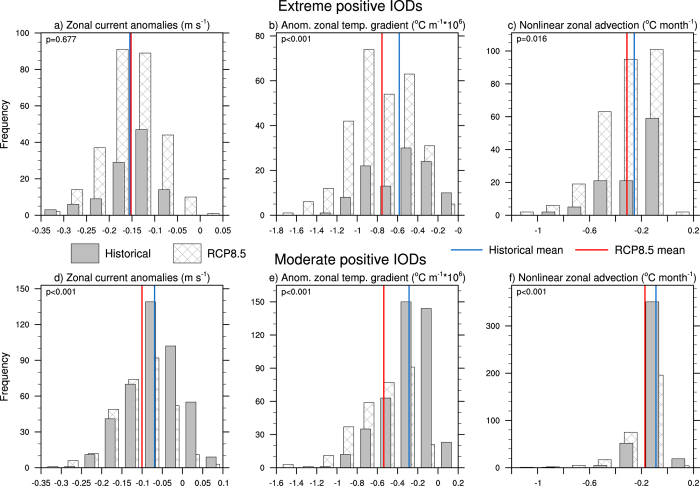
Multi-model statistics for nonlinear zonal advection and its terms. (**a**) Multi-model ensemble histogram of SON zonal current anomalies associated with extreme pIODs in the equatorial IO (60°E-100°E, 5°S-5°N). (**b**), (**c**) As in (**a**) but for the anomalous zonal temperature gradient and nonlinear zonal advection respectively. (**d**), (**e**), (**f**) As in (**a**), (**b**), (**c**) but for moderate pIODs. The solid (hatched) bars represent the historical (RCP8.5) anomalies and the solid blue (red) lines represent the historical (RCP8.5) mean values. The *p*-values from a two-tailed Student’s *t*-test are shown in the top left hand corner of each histogram. All plots were generated in NCL.

To further examine the behaviour of nonlinear zonal and vertical advection under increasing greenhouse gases, their individual components can be assessed. These are the zonal and vertical current anomalies, and the anomalous zonal and vertical temperature gradients. For zonal current anomalies that occur during extreme pIODs, it is clear that under global warming, there is an increase in the frequency for anomalies of a given magnitude (Fig. 5a, hatched bars). However despite this large increase in frequency, there is little change in the mean value between the two periods, indicating that the intensity of the anomalies is similar. In contrast, the anomalies associated with moderate pIODs display a significant shift towards more negative values in RCP8.5 and this occurs despite a reduction of moderate events in the future (Fig. 5d). This demonstrates that under global warming, it is easier to generate negative zonal current anomalies along the equator. With the anomalous zonal temperature gradient displaying a clear shift towards more negative values (i.e., warmer in the west) for both moderate and extreme events (Figs. 4b,e; changes are significant at the 95% confidence level), the tendency for more frequent occurrences of westward flow in the climate change scenario supports the increased frequency of extreme pIODs through nonlinear zonal advective cooling. The fact that the shift in the magnitude of the advective cooling is more significant for moderate pIODs than for extreme pIODs (Figs. 5c,f) illustrates the tendency for moderate events to be transformed into extreme events under greenhouse warming. This effect is also depicted in the nonlinear vertical advection as described below.

**Figure 6:**
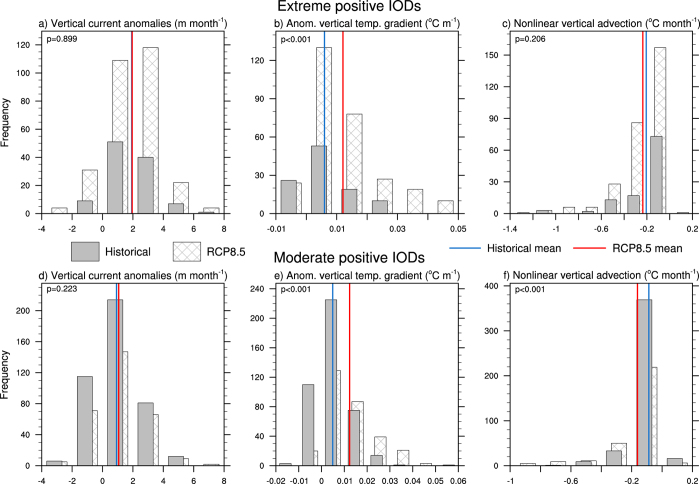
Multi-model statistics for nonlinear vertical advection and its terms. (**a**) Multi-model ensemble histogram of SON vertical current anomalies associated with extreme pIODs in the equatorial IO (60°E-100°E, 5°S-5°N). (**b**), (**c**) As in (**a**) but for the anomalous zonal temperature gradient and nonlinear zonal advection respectively. (**d**), (**e**), (**f**) As in (**a**), (**b**), (**c**) but for moderate pIODs. The solid (hatched) bars represent the historical (RCP8.5) anomalies. The solid blue (red) lines represent the historical (RCP8.5) mean values. The *p*-values from a two-tailed Student’s *t*-test are shown in the top left hand corner of each histogram. All plots were generated in NCL.

The anomalous vertical current that occurs during moderate and extreme events shows almost no change in the mean value (Figs. 6a,d), indicating that the intensity of the anomalous vertical current is not changing under global warming. In contrast the anomalous vertical temperature gradient associated with moderate and extreme pIOD events displays a strong and significant shift towards more positive values (Figs. 6b,e), meaning that it becomes stronger. During extreme pIODs, nonlinear vertical advection displays a weak tendency for more negative values in the RCP8.5 simulation but it is not significant at the 95% confidence level (Fig. 6c). However for moderate pIOD events (Fig. 6f), there is a significant shift towards stronger negative values and a distinguishable change in the size of the average anomaly. As such, the nonlinear vertical advection term associated with moderate pIODs becomes more extreme under global warming. This provides strong cooling in the eastern IO and this helps to drive the positive feedback along the equator by reinforcing the anomalous zonal temperature gradient.

### Cause of response to greenhouse warming

The changes that are observed in the anomalous ocean currents and temperature gradients are due to changes in the mean state of the tropical IO. In response to increasing greenhouse gases, the Walker circulation over the IO weakens and the western IO warms faster than the east, a response that many models simulate^22^,^23^,^24^,^25^ (Fig. S7). Due to this warming pattern, westward shifts of convection can occur more readily as a relatively smaller temperature anomaly is needed and easterly (i.e., negative) zonal wind anomalies can develop more frequently (Fig. S8). This increased frequency of zonal wind stress anomalies means that easterly zonal current and upward (i.e., positive) vertical current anomalies can also occur more often. For the anomalous temperature gradients, the tropical IO warming pattern strengthens the anomalous negative zonal temperature gradient and because the ocean surface warms faster than at depth under global warming, the anomalous vertical temperature gradient becomes stronger. The westward shift in convection also gives rise to the positive feedback along the equator involving upwelling Kelvin waves^8^. This positive feedback reinforces the nonlinear zonal and vertical advection processes and thus the development of extreme pIODs can occur more frequently.

In contrast, the damping of nIODs by NDH weakens under global warming but the other heat budget terms do not change noticeably (Fig. S4). This weaker damping is also a response to mean state change as the weaker Walker circulation reduces the anomalous westerly winds that occur during nIODs. Therefore, the anomalous zonal and vertical currents are also decreased and their associated cooling rates are weaker. This is particularly noticeable for nonlinear vertical advection (Fig. S6) and this weaker cooling may allow the equatorial IO to warm during nIODs in the future (Figs. S3c,d). Despite this, there is no considerable change in the frequency of moderate or strong nIODs (Table S1) as the mean state change makes it more difficult for strong nIOD events to occur. This response highlights the importance of NDH for extreme pIODs.

### Summary

An ensemble of 19 CMIP5 models shows that nonlinear zonal and vertical advection are important processes during extreme pIODs, yet under increasing greenhouse gases, the amplitude of these two processes does not change considerably. Examination of the two nonlinear terms and their individual components reveals that the increased occurrence of extreme events is largely due to changes in the mean state. In a warmer climate, the western tropical IO warms faster than the eastern IO. This means that the temperature anomalies required to shift convection westward are relatively smaller in the future and therefore, it becomes easier for these shifts to occur and so the frequency of their occurrence increases. During extreme pIOD events, nonlinear zonal and vertical advections stimulate a positive feedback process along the equator that reinforces the convection shift. This leads to stronger anomalous easterly winds which in turn drive upwelling equatorial Kelvin waves that increase upwelling off the Sumatra-Java coast. As the eastern IO cools further, the western tropical IO thermocline deepens, generating a stronger zonal temperature gradient and therefore, stronger anomalous easterly winds. Due to the weakening of the Walker circulation under greenhouse warming, the frequency of easterly wind anomalies increases, which in turn allows easterly zonal and upward vertical current anomalies to occur more often. This behaviour, combined with the positive feedback from westward convection shifts, means that moderate pIOD events become more extreme and the frequency of extreme pIODs increases in a warmer climate.

## Methods

### Definition of extreme IOD events

Extreme pIODs are identified using Empirical Orthogonal Function (EOF) analysis which is performed on the historical and RCP8.5 concatenated rainfall anomalies over the tropical IO^8^ (60°-110°E, 10°S-10°N). As the IOD peaks during austral spring (September-November, SON), the EOF analysis is calculated for this season. The first EOF pattern (EOF1) displays a rainfall reduction over the eastern IO and increased rainfall over the western IO. This is the typical rainfall pattern associated with moderate pIOD events. The second EOF pattern (EOF2) shows reduced rainfall further west. The relationship between EOF1 and EOF2 is nonlinear such that the combination of the two is large for more positive EOF1 and small for moderate and negative values of EOF1. As such, an extreme pIOD is defined when the EOF1 time series (i.e. the first principal component, PC1) is greater than one standard deviation and the EOF2 time series (PC2) is greater than 0.5 standard deviations^8^. This nonlinear relationship between EOF1 and EOF2 means that strong nIOD events cannot be defined using this approach. For defining strong nIODs, the detrended Dipole Mode Index^1^ (DMI) is used and a strong nIOD event is defined as when the DMI is less than −1.25 standard deviations. Moderate pIODs are when the detrended DMI is greater than 0.75 standard deviations but is not an extreme event. Similarly, moderate nIODs are when the detrended DMI is less than −0.75 standard deviations but is not a strong event. The DMI is defined as the SSTA difference between the western pole (50°E-70°E, 10°S-10°N) and the eastern pole (IODE; 90°E-110°E, 10°S-Eq.).

### Model data

Historical and RCP8.5^26^ zonal wind stress, precipitation, ocean temperature, and ocean velocities from 19 CMIP5^27^ models are used in this analysis. Only the one realisation and experiment are used for each model (i.e., r1i1p1). These 19 models (Table S1) were chosen as they are able to simulate negative IODE SST skewness and the nonlinear relationship between the rainfall EOF1 and EOF2 time series. The periods analysed each span 95-years, 1911–2005 and 2006–2100 for the historical and RCP8.5 simulations, respectively. The number of extreme pIODs varies between models and to take this into consideration, each model’s composite of extreme pIODs is used to represent the individual model’s behaviour during extreme events. This gives 19 model composites (of extreme pIODs) which are then averaged to create the multi-model ensemble mean. This also applies to moderate pIODs. Generally, most CMIP5 models simulate stronger SST variability over the IODE region and the IOD amplitude tends to be larger compared to observations^28^. Simulated biases in the IOD have been well documented, with CMIP5 models consistently simulating an eastward shoaling thermocline slope^29^, a stronger than observed negative zonal SST gradient, and equatorial easterlies that are too intense^14^. A westward extension bias of SSTAs is also present in many models. Although these biases impact the reliability of climate projections for the surrounding regions, the agreement amongst models of the weakening Walker circulation and the easterly wind trend suggests that the future behaviour of extreme pIODs is robust.

### Heat budget analysis

The surface heat balance of the tropical IO is expressed as:
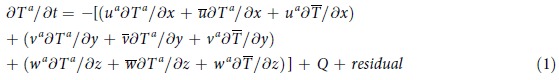
*T, u, v*, and *w* represent potential temperature, zonal, meridional, and vertical ocean current velocities respectively and are averaged over the top 50 m. Superscript ‘*a*’ denotes anomalous quantities which are referenced to the historical period. The overbar denotes historical long-term averaged quantities. Equation (1) expresses that the anomalous temperature tendency (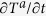) is equal to the zonal, meridional and vertical advection of temperature, the net surface air-sea heat flux (*Q*), and residual terms such as mixing and diffusion. Using a fixed depth of 50 m avoids complications associated with a multi-model ensemble such as varying mixed layer depth amongst models. Furthermore, at 50 m depth there is a strong correlation (*r* *=* 0.83) between the anomalous temperature tendency (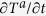) and the right-hand side of the equation (1), excluding the residual terms. This means that the residual terms are not as important for the heat budget at a depth of 50 m. Analysis using spatially and temporally varying mixed layer depth (Fig. S9) shows only small differences to the analysis using a fixed depth of 50 m (Fig. 1).

Nonlinear dynamic heating (NDH) can be described as the anomalous advection of the temperature anomaly and comprises the nonlinear terms in Equation (1). It is expressed as:
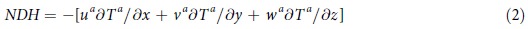


### Statistical significance

To examine whether the change between the historical and RCP8.5 periods is significant, a two-tailed Student’s *t*-test is used and values greater than the 95% confidence level are considered to be significant.

### Seasonality

All calculations in this paper are performed over the austral spring season (September-November, SON), which is when the IOD peaks. CMIP5 models are able to simulate the seasonal phase locking of the IOD well^30^.

### Graphics software

All maps and plots were produced using:

NCAR Command Language (Version 6. 3. 0) [Software]. (2015). Boulder, Colorado: UCAR/NCAR/CISL/TDD. http://dx.doi.org/10.5065/D6WD3XH5

## Additional Information

**How to cite this article**: Ng, B. *et al*. Nonlinear processes reinforce extreme Indian Ocean Dipole events. *Sci. Rep*. **5**, 11697; doi: 10.1038/srep11697 (2015).

## Supplementary Material

Supplementary Information
