# Supplementary material for: Nonlinear processes reinforce extreme Indian Ocean Dipole events
Source: Sci Rep. 2015 Jun 26;5:11697. doi: 10.1038/srep11697 (PMC4481856; doi:10.1038/srep11697)
Supplement: Supplementary Information [file srep11697-s1.pdf]

Nonlinear processes reinforce extreme Indian Ocean Dipole events

Benjamin Ng*[1,2], Wenju Cai[1], Kevin Walsh[2], and Agus Santoso[3]

[1]CSIRO Marine and Atmospheric Research, Aspendale, Victoria, Australia.

[2]School of Earth Sciences, University of Melbourne, Parkville, Victoria, Australia.

[3]Australian Research Council Centre of Excellence for Climate System Science, University of New South Wales, Sydney, New South Wales, Australia

*Correspondence and request for materials should be addressed to B. N.

(Benjamin.Ng@csiro.au)

Supplementary tables and figures

Figures

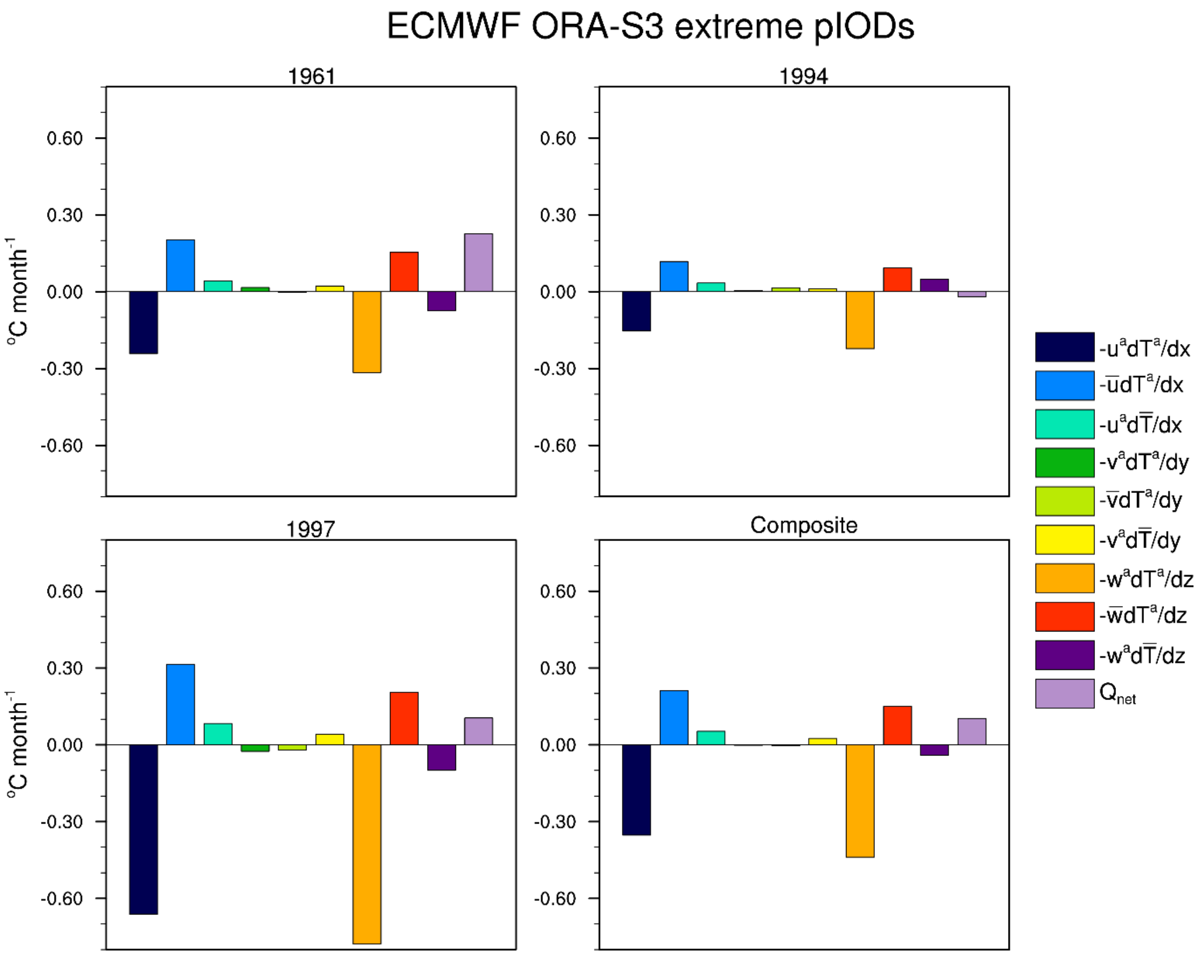

Figure S1 | Heat budget analysis of extreme pIOD events in ECMWF-ORAS3 ocean reanalysis. Bar charts of the SON heat budget components during three extreme pIOD events (1961, 1994, and 1997) and the composite of the three extreme events. Table S2 provides a description of each heat budget term.

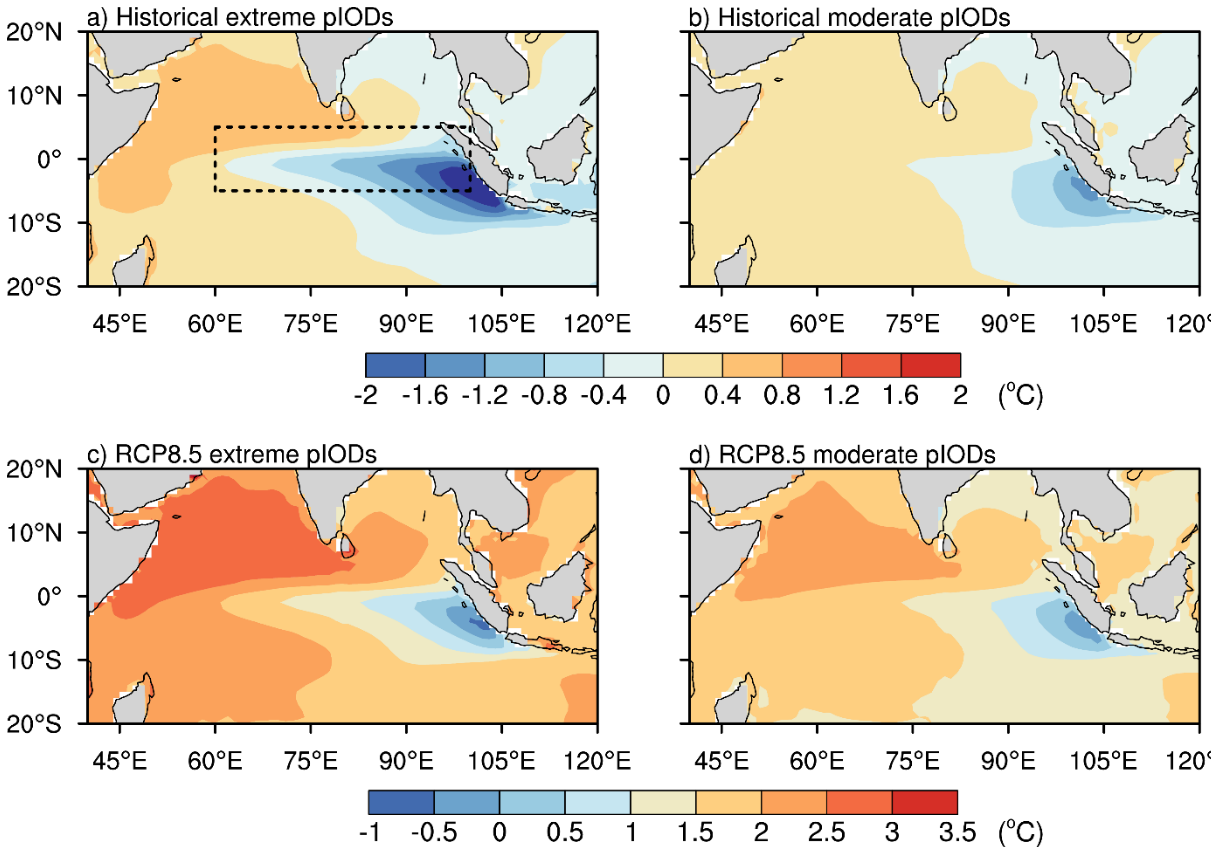

Figure S2 | Sea surface temperature anomaly during extreme and moderate pIOD events. (a) Map showing the multi-model ensemble mean historical (1911-2005) SON sea surface temperature anomaly during extreme pIODs. (b) As in (a) but for moderate pIODs. (c), (d) As in (a), (b) respectively but for the RCP8.5 period (2006-2100). The dashed black box in (a) marks the equatorial IO region (60°E-100°E, 5°S-5°N).

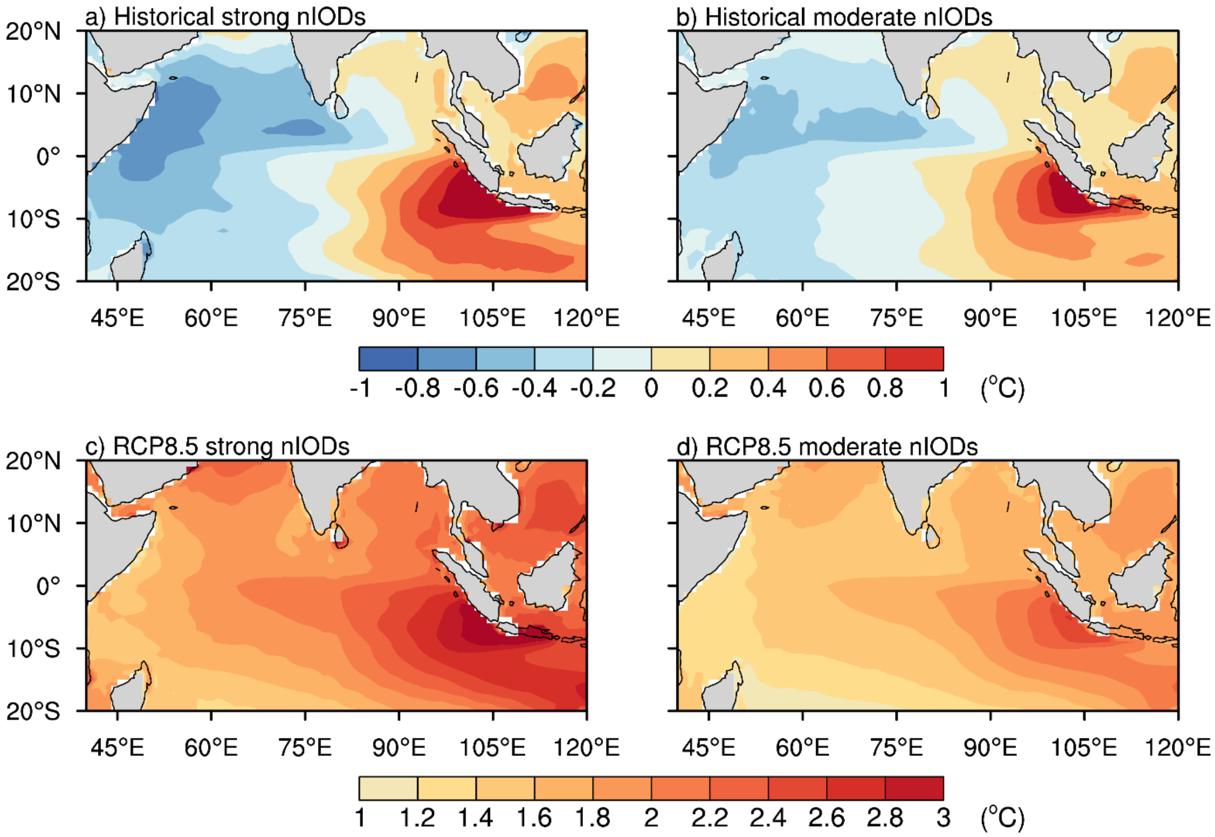

Figure S3 | Sea surface temperature anomaly during strong and moderate negative IOD (nIOD) events. (a) Map showing the multi-model ensemble mean historical SON sea surface temperature anomaly during strong nIODs. (b) As in (a) but for moderate nIODs. (c), (d) As in (a), (b) respectively but for the RCP8.5 period. A strong nIOD is when the DMI is less than -1.25 standard deviations. A moderate nIOD is when the DMI is less than -0.75 standard deviations but is not a strong event.

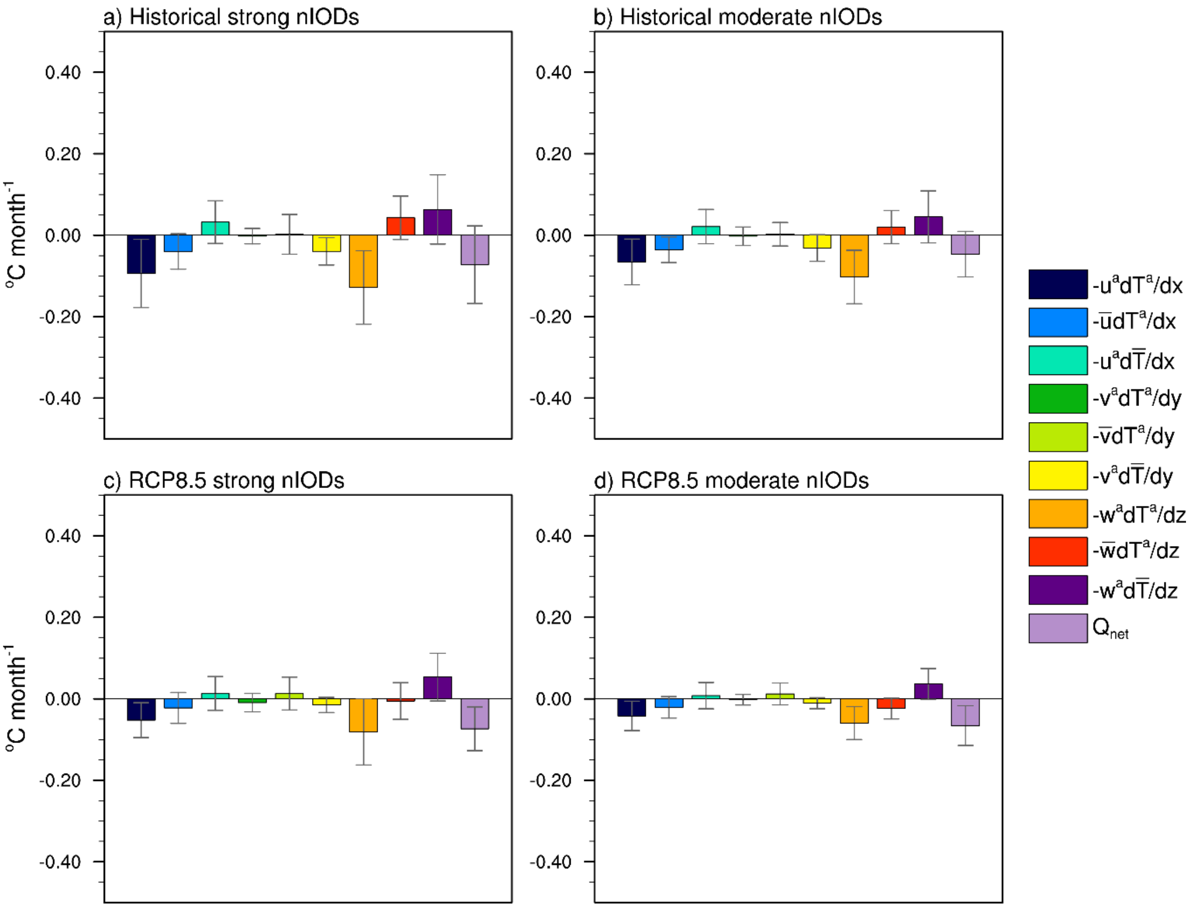

Figure S4 | Heat budget analysis of negative IOD (nIOD) events in CMIP5 models. (a) Multi-model ensemble averaged historical SON heat budget components for strong nIODs. (b) As in (a) but for moderate nIODs. (c), (d) As in (a), (b) respectively but for the RCP8.5 period. Table S2 provides a description of each heat budget term.

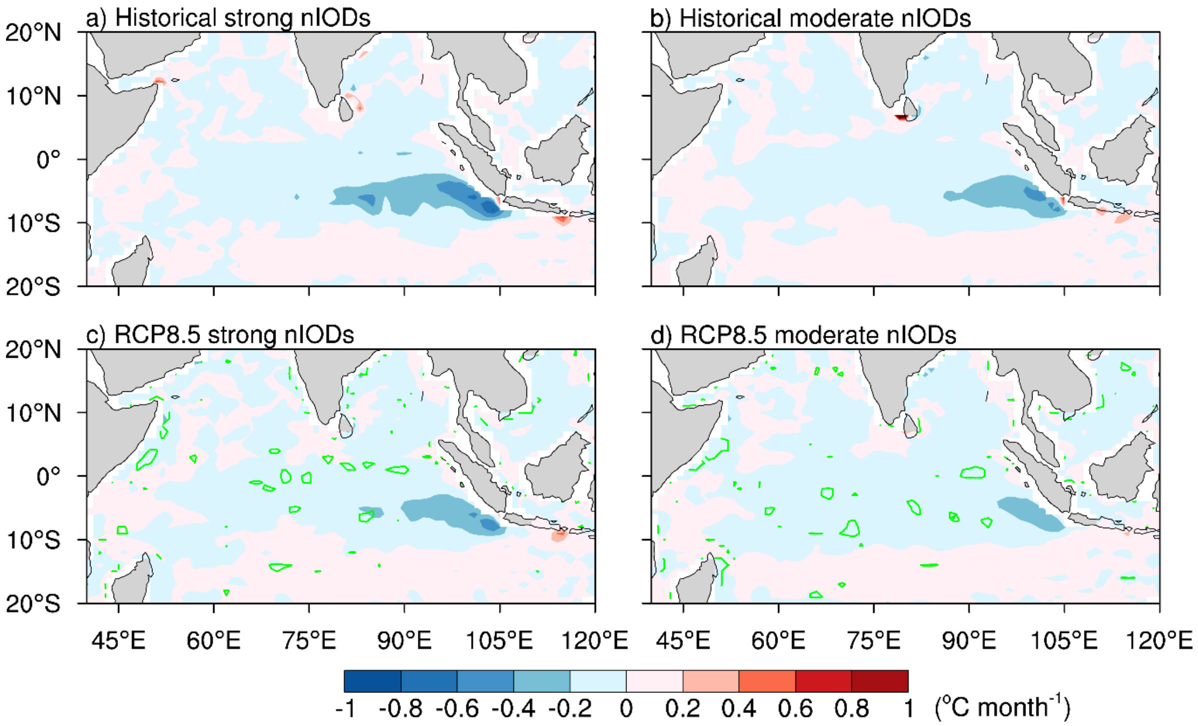

Figure S5 | Nonlinear zonal advection during strong and moderate nIOD events. (a) Map showing the multi-model ensemble mean historical SON nonlinear zonal advection term during strong nIODs. (b) As in (a) but for moderate nIODs. (c), (d) As in (a), (b) respectively but for the RCP8.5 period. The green contours in (c) and (d) denote where the difference between the historical and RCP8.5 periods is significant at the 95% confidence level based on a two-tailed Student's *t*-test.

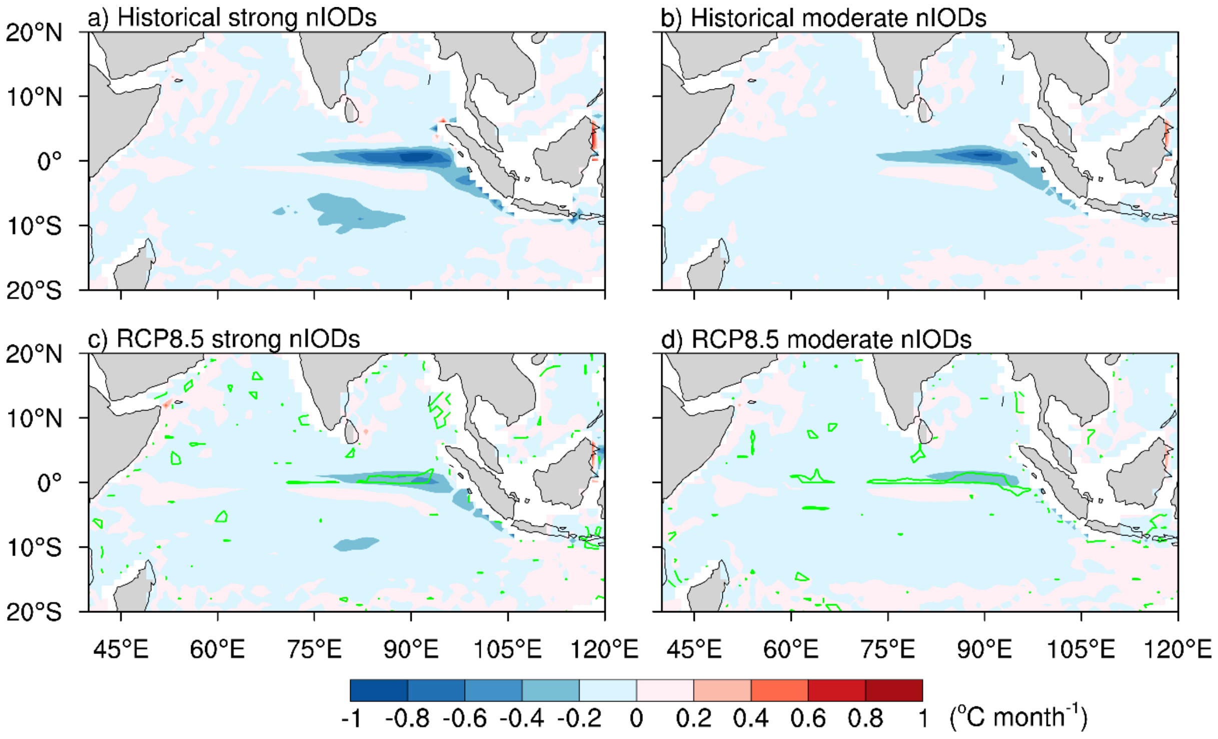

Figure S6 | Nonlinear vertical advection during strong and moderate nIOD events. (a) Map showing the multi-model ensemble mean historical SON nonlinear vertical advection term during strong nIODs. (b) As in (a) but for moderate nIODs. (c), (d) As in (a), (b) respectively but for the RCP8.5 period. The green contours in (c) and (d) denote where the difference between the historical and RCP8.5 periods is significant at the 95% confidence level based on a two-tailed Student's *t*-test.

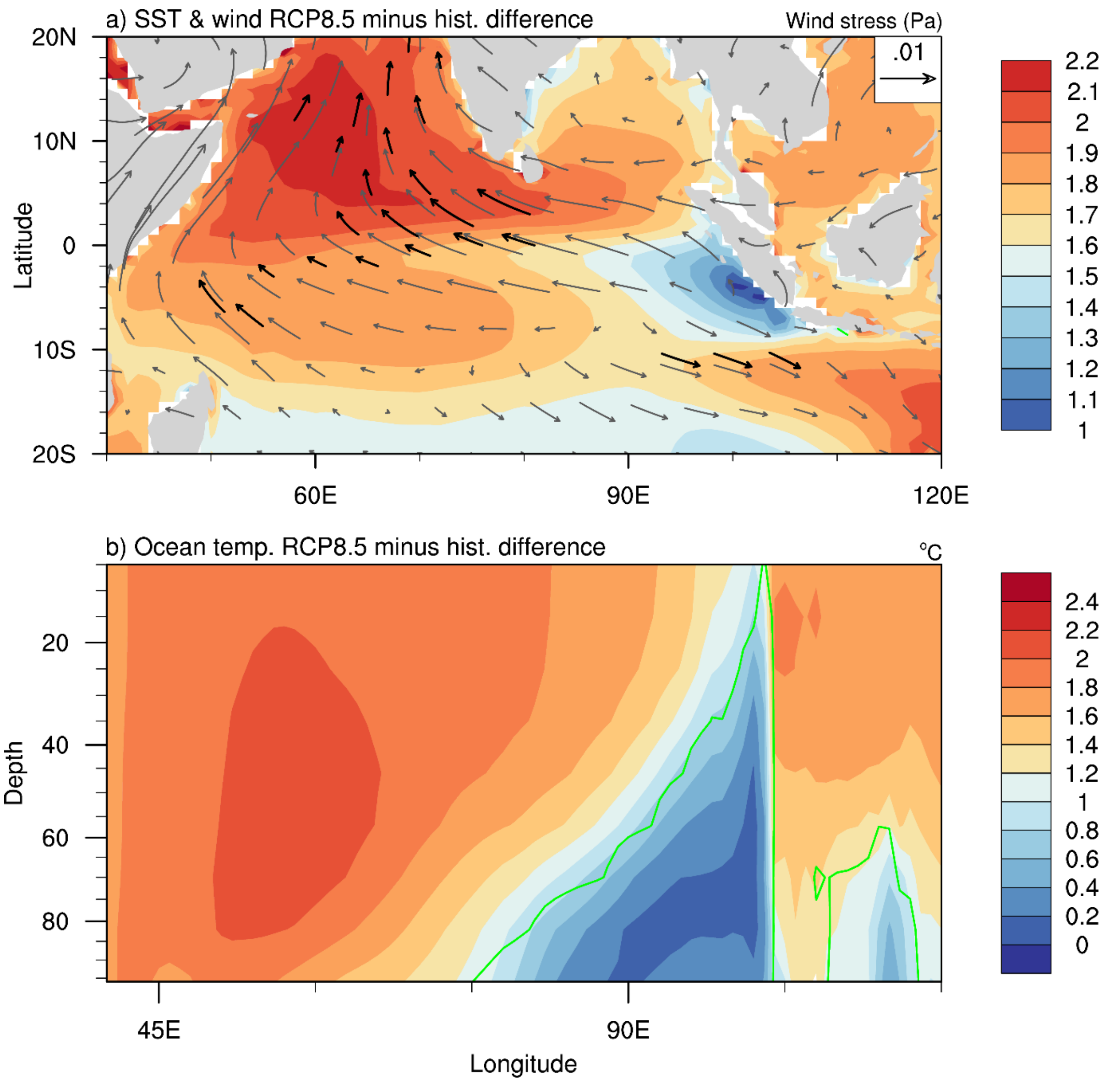

Figure S7 | Mean state change over the tropical Indian Ocean. (a) Map showing the multi-model ensemble SON RCP8.5 minus historical difference in SST (colours) and wind stress (vectors). The change in SST is significant everywhere, black vectors are where the zonal or meridional wind stress change is significant at the 95% confidence level. (b) Depth versus longitude plot of the multi-model ensemble SON RCP8.5 minus historical difference in ocean temperature. The green contours denote where the difference between the two periods is significant at the 95% confidence level based on a two-tailed Student's *t*-test.

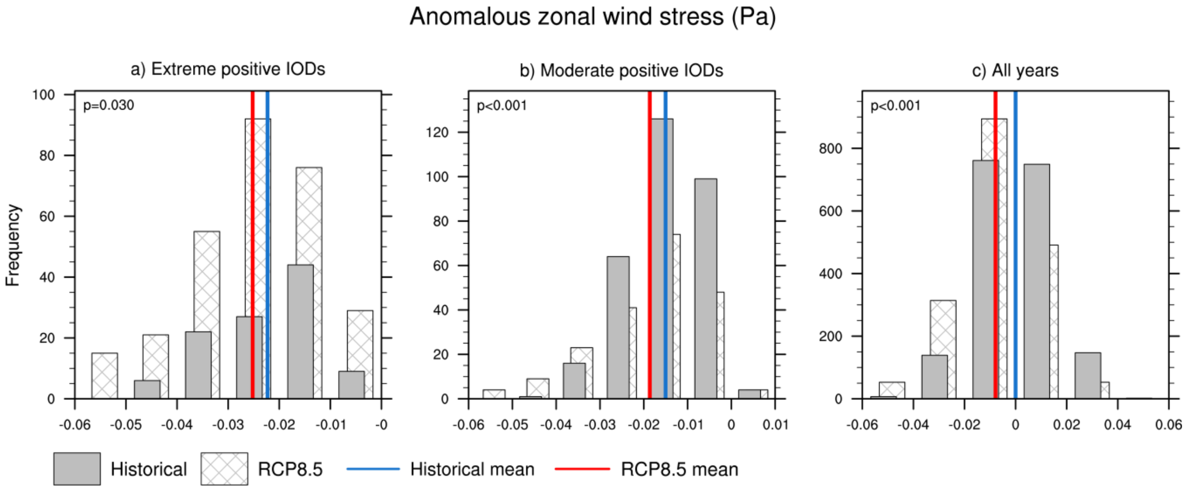

Figure S8 | Multi-model statistics for zonal wind stress. (a) Multi-model ensemble histogram of SON zonal wind stress anomalies associated with extreme pIODs in the equatorial IO (60°E-100°E, 5°S-5°N). (b), (c) As in (a) but for moderate pIODs and all years respectively. The solid (hatched) bars represent the historical (RCP8.5) anomalies. The solid blue (red) lines represent the historical (RCP8.5) mean values. The p-values from a two-tailed Student's t-test are shown in the top left hand corner of each histogram.

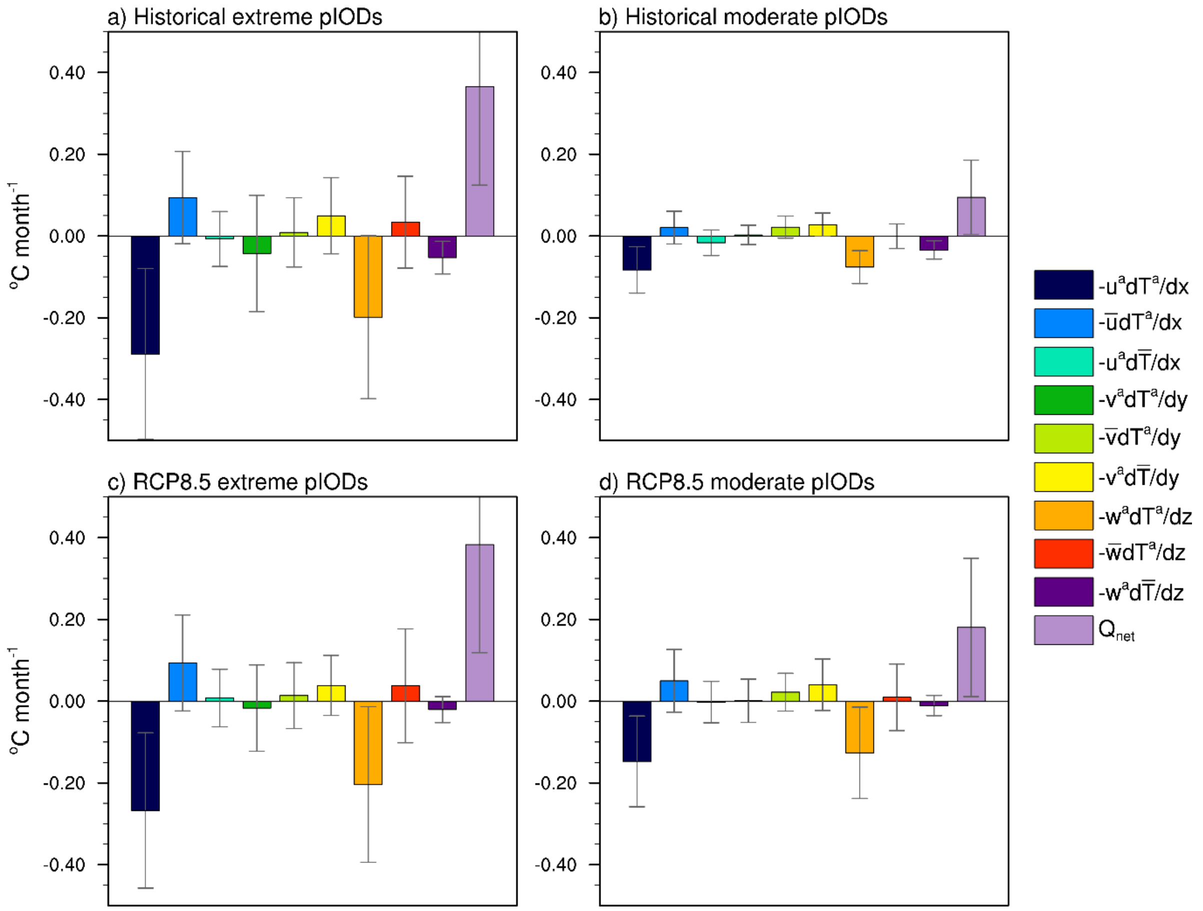

Figure S9 | Multi-model ensemble mean heat budget analysis of extreme and moderate pIOD events using spatially and temporally varying mixed layer depth. (a) Multi-model ensemble averaged historical (1911-2005) SON heat budget components for extreme pIODs. (b) As in (a) but for moderate pIODs. (c), (d) As in (a), (b) respectively but for the RCP8.5 period (2006-2100). The error bars indicate 1 standard deviation of the multi-model ensemble. The heat budget components are averaged over the equatorial IO (60°E-100°E, 5°S-5°N). The mixed layer depth is defined as the density at 10 m depth plus an increment in density equivalent to a 0.2°C cooling. Compared to ECMWF ORA-S3 reanalysis, the multi-model ensemble mixed layer depth is simulated reasonably well (not shown).

Tables

Model	Hist. IODE SST skewness	Moderate pIODs (Hist./RCP8.5)	Extreme pIODs (Hist./RCP8.5)	Moderate nIODs (Hist./RCP8.5)	Strong nIODs (Hist./RCP8.5)
ACCESS1-0	-0.15	23/13	5/14	8/7	6/7
bcc-csm1-1-m	-0.58	38/31	1/14	7/5	1/4
CanESM2	-0.75	39/29	1/22	1/6	0/1
CESM1-CAM5	-0.39	26/13	9/22	15/12	**3/3**
CMCC-CESM	-0.73	16/12	4/11	16/16	3/4
CNRM-CM5	-0.14	17/13	12/15	16/21	**12/6**
CSIRO-Mk3-6-0	-0.26	37/18	2/25	8/3	1/5
GFDL-CM3	-0.53	25/16	5/20	5/15	**2/2**
GFDL-ESM2G	-0.66	6/6	**10/7**	13/12	**8/7**
GFDL-ESM2M	-0.73	18/14	9/20	11/15	0/3
HadGEM2-CC	-0.01	23/12	7/17	7/8	**8/3**
IPSL-CM5A-LR	-0.34	26/16	5/8	8/9	**6/3**
IPSL-CM5B-LR	-0.07	14/10	8/14	20/10	9/15
MIROC5	-0.71	25/12	1/15	7/14	3/15
MPI-ESM-LR	-1.80	24/26	3/11	5/8	0/9
MPI-ESM-MR	-0.99	20/14	3/19	7/13	1/2
MRI-CGCM3	-0.59	19/19	6/12	14/12	4/9
NorESM1-M	-0.08	15/14	7/12	11/15	**10/8**
NorESM1-ME	-0.27	17/12	10/10	16/14	7/10
**Total events**	-0.45 (mean)	428/300	108/288	195/215	84/116

Table S1 | Number of IOD events for each model. Table showing the historical IODE SST skewness (1st column), the number of moderate pIOD events (2nd column), the number of extreme pIOD events (3rd column), the number of moderate nIOD events (4th column), and the number of strong nIOD events (5th column). The bold blue type indicates models that have a reduction in extreme pIOD events. The red type indicates models that have a decrease or no change in strong nIOD events. These models were selected based on their ability to simulate negative IODE SST skewness in the historical period (1911-2005) as well as the nonlinear relationship between rainfall EOF1 and EOF2.

Heat budget term	Description
$\frac{\partial T^a}{\partial t}$	The change in anomalous ocean temperature over time.
$u^a \frac{\partial T^a}{\partial x}, v^a \frac{\partial T^a}{\partial y}, w^a \frac{\partial T^a}{\partial z}$	Anomalous zonal, meridional, and vertical advection of the anomalous ocean temperature, respectively. This can be thought of as the transport of anomalous heat by anomalous ocean currents. Collectively, these nonlinear advection terms form the nonlinear dynamic heating (NDH) process where: $NDH = -\left(u^a \frac{\partial T^a}{\partial x} + v^a \frac{\partial T^a}{\partial y} + w^a \frac{\partial T^a}{\partial z}\right)$
$\bar{u} \frac{\partial T^a}{\partial x}, \bar{v} \frac{\partial T^a}{\partial y}, \bar{w} \frac{\partial T^a}{\partial z}$	Mean zonal, meridional, and vertical advection of the anomalous ocean temperature, respectively. This can also be described as the transport of anomalous heat by the mean ocean currents.
$u^a \frac{\partial \bar{T}}{\partial x}, v^a \frac{\partial \bar{T}}{\partial y}, w^a \frac{\partial \bar{T}}{\partial z}$	Anomalous zonal, meridional, and vertical advection of the mean ocean temperature, respectively. This is the transport of the mean heat by anomalous ocean currents.
Q	Net incoming surface air-sea heat flux.
$residual$	Residual terms such as eddy mixing and diffusion.

Table S2 | Description of each heat budget term.
